# Supplementary material for: Visible light-induced switching of soft matter materials properties based on thioindigo photoswitches
Source: Nat Commun. 2023 Dec 14;14:8298. doi: 10.1038/s41467-023-44128-8 (PMC10721821; doi:10.1038/s41467-023-44128-8)
Supplement: Supplementary file 1 — Supplementary Information [file 41467_2023_44128_MOESM1_ESM.pdf]

Supplementary Information

Visible Light-Enabled Switching of Soft Material Properties Based on Thioindigo Photoswitches

Sarah L. Walden,[a, b] Phuong H.D. Nguyen,[c] Hao-kai Li,[d] Xiaogang Liu,[d] Minh T.N. Le,[c] Xian Jun Loh,*[e, f] Christopher Barner-Kowollik,*[a,b,g] Vinh X. Truong*[b, e]

[a] Centre for Materials Science, Queensland University of Technology, 2 George Street, Brisbane, QLD 4000, Australia.

[b] School of Physics and Chemistry, Queensland University of Technology, 2 George Street, Brisbane, QLD 4000, Australia.

[c] Department of Pharmacology and Institute for Digital Medicine, Yong Loo Lin School of Medicine, National University of Singapore, Singapore 117600, Republic of Singapore.

[d] Fluorescence Research Group, Singapore University of Technology and Design, 8 Somapah Road, 487372 Singapore, Republic of Singapore.

[e] Institute of Materials Research and Engineering (IMRE), Agency for Science, Technology and Research (A*STAR), 2 Fusionopolis Way, Singapore 138634, Republic of Singapore.

[f] Institute of Sustainability for Chemicals, Energy and Environment (ISCE2), Agency for Science, Technology and Research (A*STAR), 1 Pesek Road, Jurong Island, Singapore 627833, Republic of Singapore.

[g] Institute of Nanotechnology (INT), Karlsruhe Institute of Technology (KIT), Hermann-von-Helmholtz-Platz 1, 76344 Eggenstein-Leopoldshafen, Germany

*Correspondence to Xian Jun Loh (lohxj@imre.a-star.edu.sg) , Christopher Barner-Kowollik (christopher.barnerkowollik@qut.edu.au) , or Vinh X. Truong (vinh_truong@isce2.a-star.edu.sg).

1. Supplementary Methods

1.1 Materials

2,2-dithiodisalicylic acid (95%), sodium dithionite (>85%), chloroacetic acid (99%), $AlCl_3$ (99%), 2-Hydroxyethyl methacrylate (97%), 3-mercaptopropionic acid (99%), propiolic acid (95%), Poly(ethylene glycol) (BioUltra, 2000) and other chemicals (reagent grade) were obtained from Sigma-Aldrich and used as received. 4arm PEG 20,000 Da was purchased from Jenkem Tech, USA. Triethylamine (>99.0%) and thionyl chloride (98%) were purchased from TCI. Solvents (methanol, ethanol, DMF, THF, DMSO, diethyl ether, dichloromethane and chloroform) were obtained from VWR international in ACS reagent grade. Reagents used for cell culture and analysis were specified in the Cell Cytotoxicity Testing section.

1.2 NMR Spectroscopy

^{1}H and 13C NMR spectra were recorded on a JEOL (Tokyo, Japan) ECA 400 II spectrometer at ambient temperature. $CDCl_3$ (Cambridge Isotope Laboratories, USA) were used as the solvents for the NMR analysis, and the chemical shift was calibrated using residual non-deuterated solvents or tetramethylsilane (TMS) as the internal standard.

1.3 UV-Vis Spectroscopy

UV-Vis spectra were recorded on a Shimadzu UV-2700 spectrophotometer equipped with a CPS-100 electronic temperature control cell positioner. For molar absorptivity measurements, stock solutions of the samples were prepared in chloroform. Aliquots were added to a 2 mL solution of chloroform in Thorlabs UV Fused Quartz Cuvettes (CV10Q35F) to obtain five measurements of absorbance between 0 and 1 absorbance units. Spectra were measured at 25 °C.

1.4 THF-Size Exclusion Chromatography

SEC analysis was performed on a Shimadzu P-Series liquid chromatograph LC-20AD equipped with a Shodex LF-804 mixed gel column (300 × 8.0 mm; bead size = 6 μm; pore size = 3000 Å) using THF as an eluent. The flow rate was 1 mL·min (40 °C). The sample detection was conducted using a refractive index detector (RID-20A). The column system was calibrated with polystyrene standard.

1.5 Details of In-situ Absorbance for Photoisomerisation Action Plot

The on-line tracking of absorbance spectra during irradiation was conducted using an in-situ UV-Vis apparatus depicted in **Fig. S1**. An Ocean Optics DH-MINI Deuterium-Tungsten-Halogen lamp was

coupled via optic fibres (P400-025-SR) to an Ocean Optics FLAME-T-UV-VIS spectrometer, sensitive from 200 to 850 nm, via a cuvette holder.

A custom designed cuvette holder was enabled laser irradiation into the bottom of the cuvette, while simultaneously measuring absorbance through the side of the cuvette. An Opolette 355 tuneable OPO, emitting 5 ns pulses from 210-2400 nm at a repetition rate of 20 Hz, was directed upwards into the bottom of a quartz fluorescence (Hellma Analytics quartz high precision cell). The laser energy deposited into the sample was measured above the aluminum block before and after experiments using a Coherent EnergyMax thermopile sensor (J-25MB-LE) to account for any power fluctuations during irradiation. Kinetic absorbance data at 540 nm was recorded every 250 ms (50 ms integration time, 5 scan average). In additional, full spectra were saved every 10 s during irradiation. All data processing was performed in Matlab®.

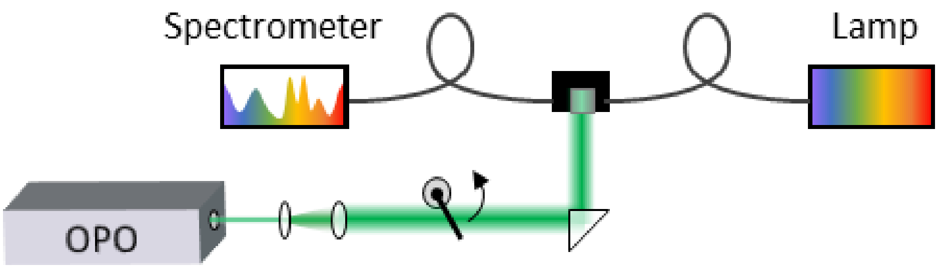

Supplementary Figure 1. Schematic diagram of experimental apparatus for the photophysical studies. The tuneable OPO output is passed through beam expansion optics before being incident on the bottom of a quartz fluorescence cuvette. Perpendicular, fibre-coupled white light is passed through the cuvette and the absorbance spectrum is captured on a fibre coupled spectrometer.

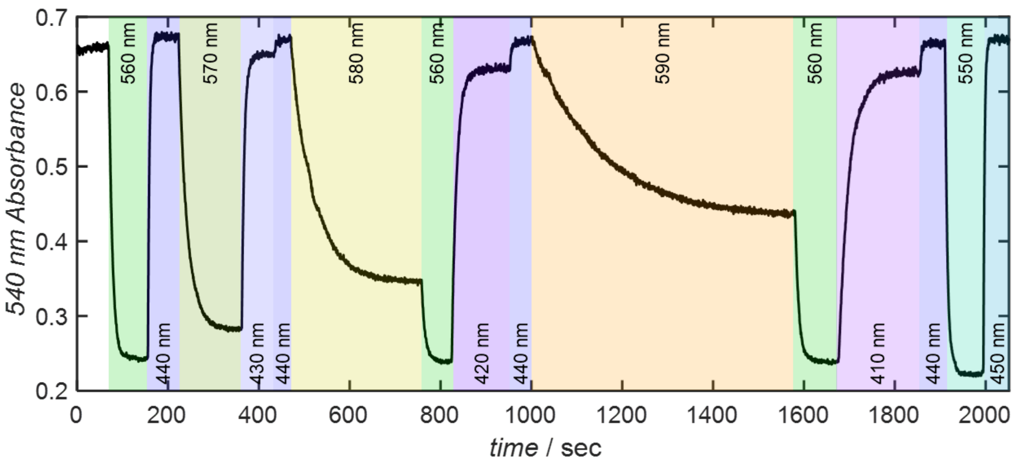

Supplementary Figure 2. Typical 540 nm absorption kinetic data recorded for during photoisomerization of thioindigo **1** action plot measurements. Absorbance at 540 nm was captured

every 250 ms during irradiation from below with various wavelengths from a tuneable nanosecond OPO.

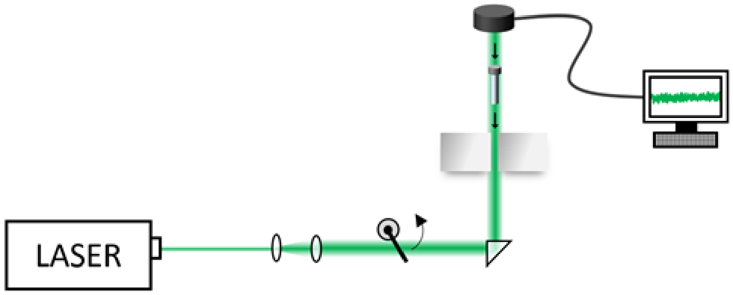

Supplementary Figure 3. Schematic diagram of apparatus used for laser experiments.

The recorded data was imported into matlab and exponential fits were performed to the data captured during irradiation with each wavelength according to the following formula

$$a \exp[k(t - t_0)] + c \quad (1a)$$

$$a\{1 - \exp[k(t - t_0)]\} + c \quad (1b)$$

Where *a* is a proportionality constant, *k* is the switching rate in s^{-1}, t_0 is the time offset and *c* is the absorbance plateau value used to determine the cis-/trans- ratio. Equations 1a and 1b were applied to the *trans*- to *cis*- and *cis*- to *trans*- isomerisations, respectively.

Once this switching rate at each wavelength was determined, the next step was to normalise the switching rate by the incident photon flux using the relation:

$$k_{norm} = \frac{k}{(E_{pulse} f T_\lambda / E_\lambda)} \quad (2)$$

Where k_{norm} is the normalised switching rate in units of photons^{-1}, E_{pulse} is the measured pulse energy incident on the cuvette, f is the frequency of the laser pulses, T_λ is the wavelength dependent cuvette transmission and $E_\lambda = hc/\lambda$ is the photon energy at each wavelength.

1.6 Quantum Yield Determination

The reaction quantum yields were determined by measuring kinetics of the photoswitching during irradiation and performing least squares fits of the experimentally determined conversions to the library of theoretical conversions simulated in Matlab®, (Eqns 3a-3b) at each time interval. For a range of different values of the photoisomerization quantum yields, Φ_{trns} *and* Φ_{cis}, using the starting

concentration $c(t_0)$, the molar absorptivity ε, the pulse energy E_{pulse} and the wavelength λ, the conversion after each laser pulse (n) was determined using the previous time step (n-1) by

$$c_{trns}(t_n) = c_{tr}(t_{n-1}) - \frac{E_{pulse}\lambda}{hc}\,\phi_{trns}\,10^{-\epsilon_{trns}c_{trns}(t_{n-1})L} + \frac{E_{pulse}\lambda}{hc}\,\phi_{cis}\,10^{-\epsilon_{cis}c_{cis}(t_{n-1})L} \quad (3a)$$

$$c_{cis}(t_n) = c_{cis}(t_{n-1}) + \frac{E_{pulse}\lambda}{hc}\,\phi_{trns}\,10^{-\epsilon_{trns}c_{trns}(t_{n-1})L} - \frac{E_{pulse}\lambda}{hc}\,\phi_{cis}\,10^{-\epsilon_{cis}c_{cis}(t_{n-1})L} \quad (3b)$$

Given the long irradiation times, diffusion was not taken into account in the simulation. A library of simulations, with quantum yield values varying between 0 and 1, was generated for each sample. Least squares fitting of the simulation library to the experimental data was subsequently performed to determine the best fit simulation, and thus quantum yields.

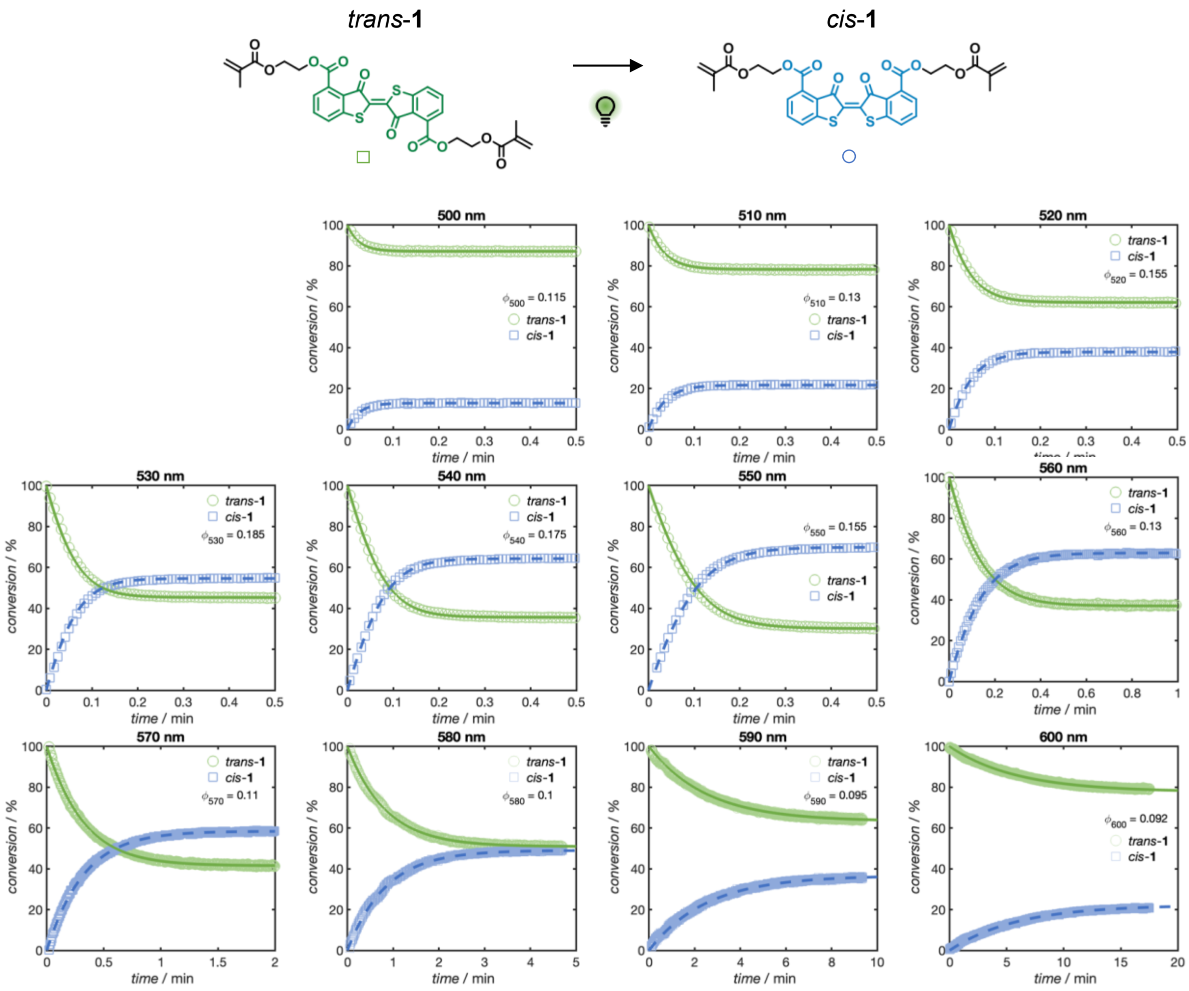

Supplementary Figure 4. Plots of experimental data measured during the conversion of *trans*-**1** (green circles) to *cis*-**1** (blue squares) during irradiation with wavelengths indicated in the figure legends. Solid lines show best fit simulation of *trans*-**1** (solid green) and *cis*-**1** (dashed blue) and the corresponding quantum yield value.

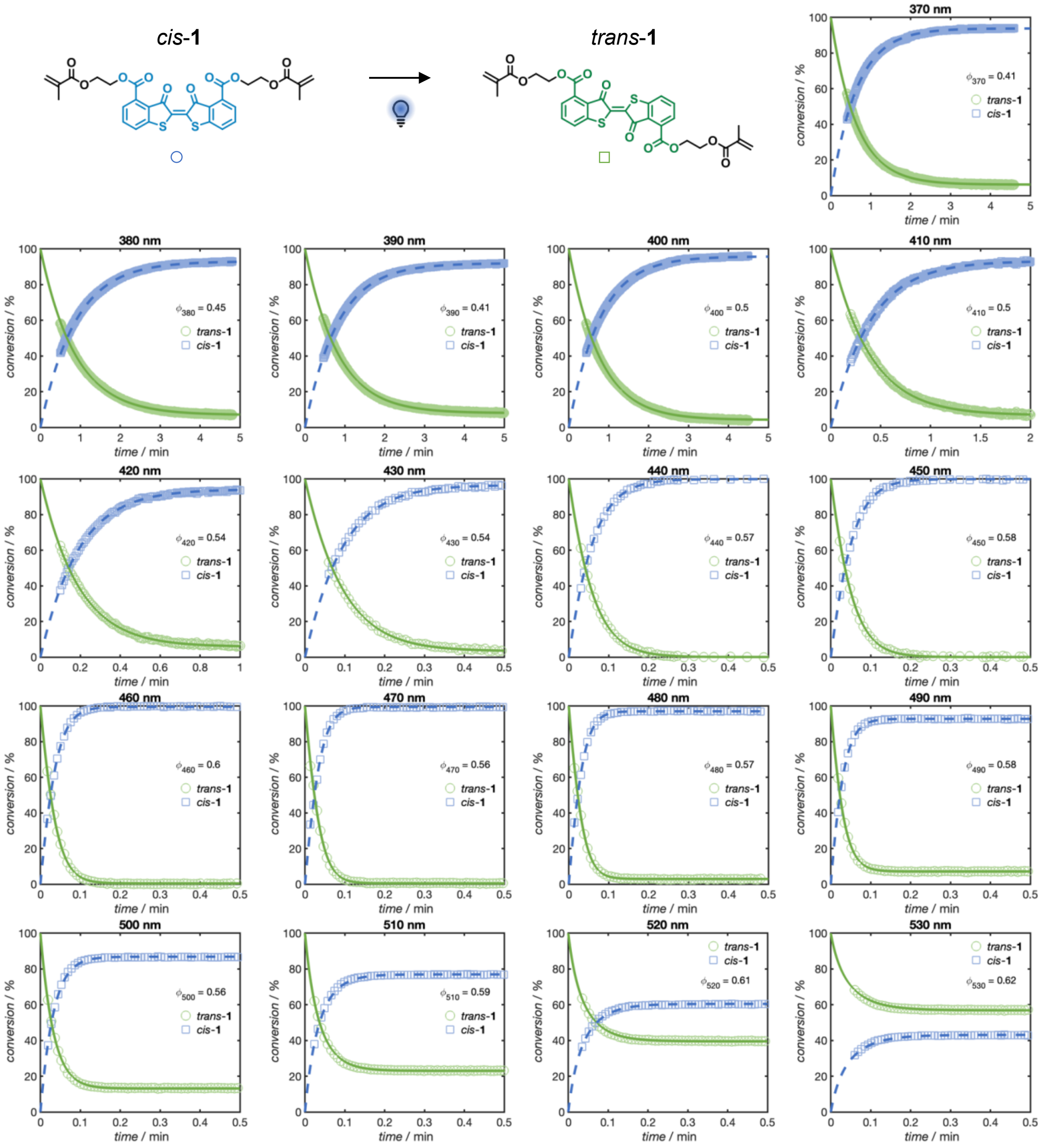

Supplementary Figure 5. Plots of experimental data measured during the conversion of *cis*-**1** (clue squares) to *trans*-**1** (green triangles) during irradiation with wavelengths indicated in the figure legends. Solid lines show best fit simulation of *cis*-**1** (dashed blue) and *trans*-**1** (solid green) and corresponding quantum yield value.

1.6 LED Light Irradiation

Light irradiation thioindigo-containing polymer solutions and hydrogel was performed using commercial LED light with wavelengths at λ_{max}= 470 nm (blue light), and 540 nm (green light),

respectively (**Supplementary Figure 4)**. LED emission spectra were recorded using an Ocean Insight Flame-T-UV-Vis spectrometer, with an active range of 200 – 850 nm and an integration time of 10 ms.

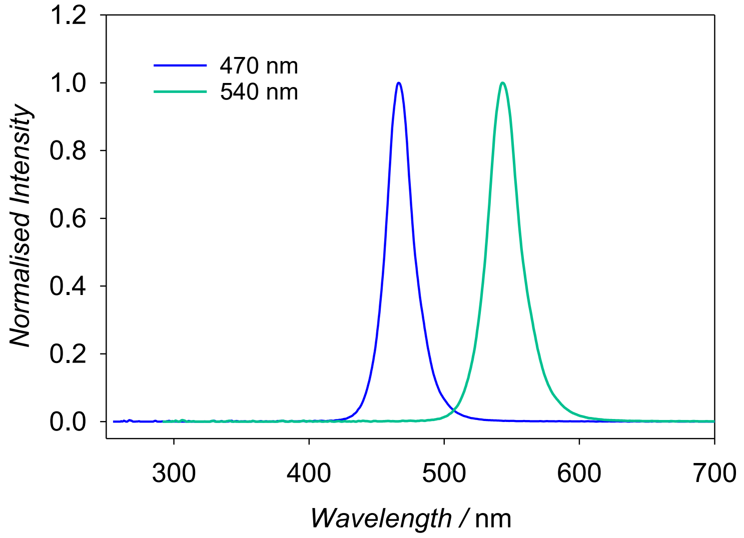

Supplementary Figure 6. Emission spectra of LED light used for irradiation.

1.7 Rheological Measurements

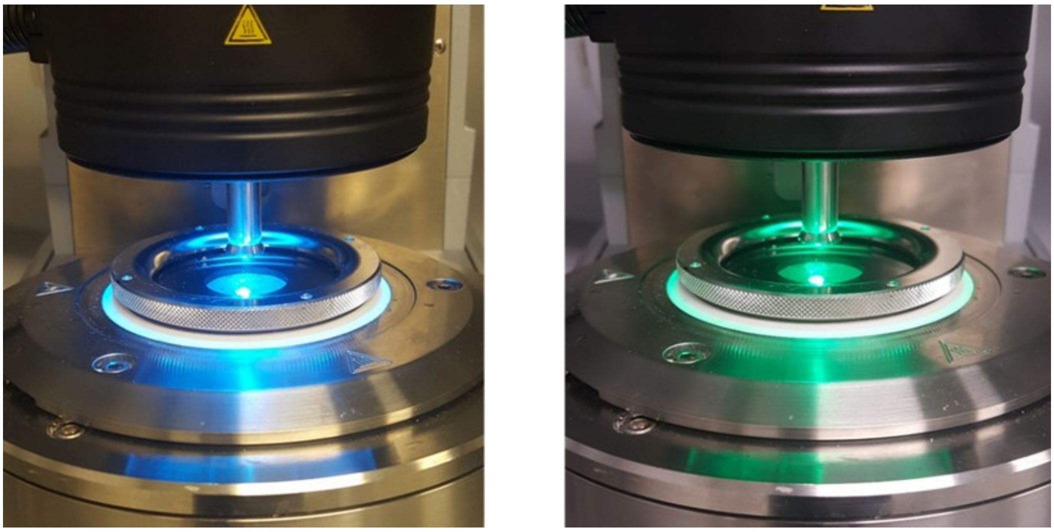

Supplementary Figure 7. Photographs of the rheometer set-up employed for following the photoswitching behaviour of thioindigo-containing hydrogels. The light source is from a wavelength-switchable device (Mightex WheeLED™), connected by a liquid light guide underneath the quartz plate.

1.8 Synthesis

Synthesis of the thioindigo-bismethacrylate (*trans*-1)

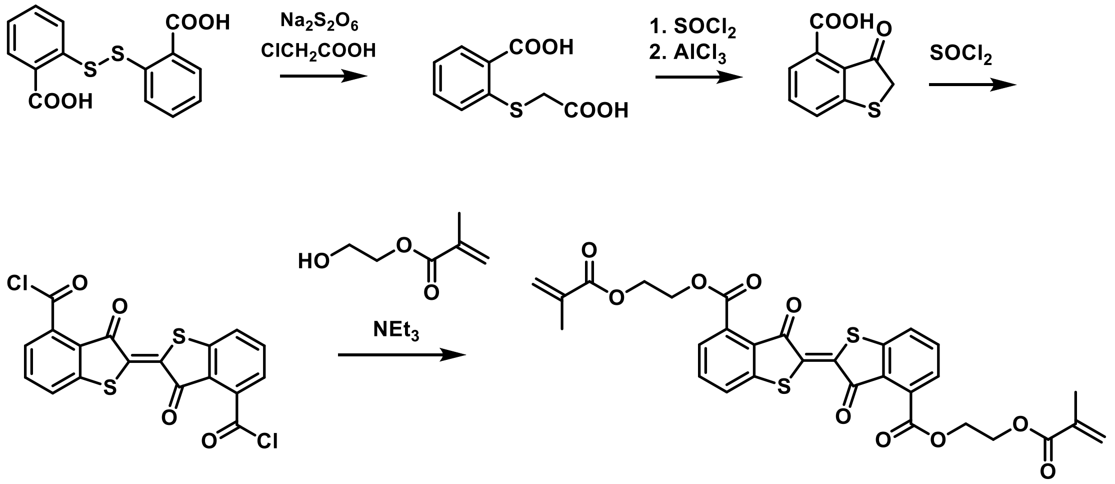

Step 1: To a solution of sodium carbonate (20 g) in water (150 mL) 2,2-dithiodisalicylic acid (10 g, 32.6 mmol) and sodium dithionite (15 g, 86.2 mmol) were added. The solution was subsequently heated at 110 °C under refluxing conditions for 30 min. In a conical flask, a solution of chloroacetic acid (15 g, 159 mmol) was neutralized with solid sodium carbonate, then added to the refluxing reaction mixture; the heating was continued for 1 h. The solution was allowed to cool to ambient temperature and acidified with concentrated hydrochloric acid to pH 1 on an ice bath. The yellow precipitate was filtered, dried and used directly in the next step.

Step 2: The above solid (8.00 g, 37.6 mmol) was dissolved in $SOCl_2$ (40 mL) and the solution was heated at 70 °C under reflux for 2 h. $SOCl_2$ was removed *in vacuo* and the residue was dissolved in 1,2-dichloroethane (50 mL) under a nitrogen blanket. The solution was cooled on an ice bath and $AlCl_3$ (9.4 g, 70.4 mmol) was added in 6 portions. The resultant green mixture was subsequently stirred under nitrogen at ambient temperature for 16 h. The reaction was quenched with ice/water and extracted with dichloromethanne (2 × 50 mL). The combined organic phase was washed with water (50 mL), brine (50 mL), dried over anhydrous $MgSO_4$, and concentrated in vacuo to give the product as an orange solid that was used directly in the next step.

Step 3: The above solid (6.5 g, 33.5 mmol) was dissolved in a solution of $SOCl_2$ (50 mL) and heated under refluxing conditions at 80 °C during which a red solid was formed. $SOCl_2$ was concentrated *in vacuo* and the solid was washed with THF (50 mL x 3). $CHCl_3$ (100 mL) was added to the mixture, followed by a solution of 2-hydroxyethyl methacrylate (4.55 g, 35 mmol) in chloroform (50 mL); the solution was cooled on an ice bath. Triethyl amine (4 g, 40 mmol) was added dropwise to the cold

solution over 30 min. The solution was allowed to warm to ambient temperature and concentrated *in vacuo*. The solid residue was washed with water (200 mL), acetone (200 mL) and dry in vacuo to give pure product as a bright red solid (3.1 g, total yield: 15.4%).

^{1}H NMR (400 MHz, $CDCl_3$, δ, ppm): 8.31-8.33 (d, *J* = 7.7 Hz), 8.12-8.14 (d, *J* = 7.7 Hz), 7.42-7.46 (t, *J* = 7.7), 6.18 (s), 5.59 (s), 4.69-4.71 (t, *J* = 4.27), 4.55-4.57 (t, *J* = 4.76), 1.95 (s).

^{13}C NMR: (101 MHz, $CDCl_3$, δ, ppm): 189.75, 167.25, 164.65, 137.26, 135.42, 130.84, 130.57, 126.46, 125.43, 18.41.

HRMS: [$C_{20}H_{14}O_{10}S_2$] Calculated: [MH^+] 609.0844, Found: [MH^+] 609.0841.

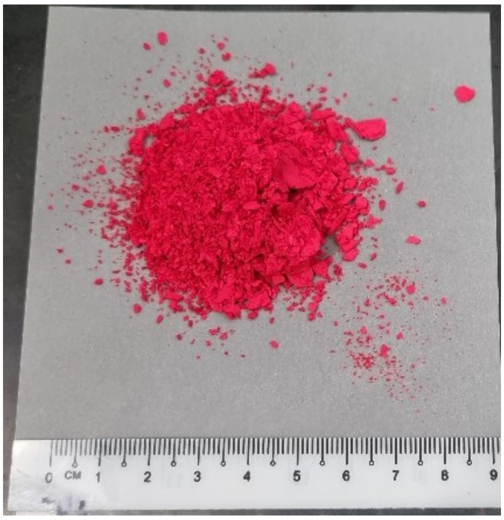

Supplementary Figure 8. Photograph of the thioindigo bismethacrylate *trans*-**1** product, which can be readily prepared in gram scale (3.1 g).

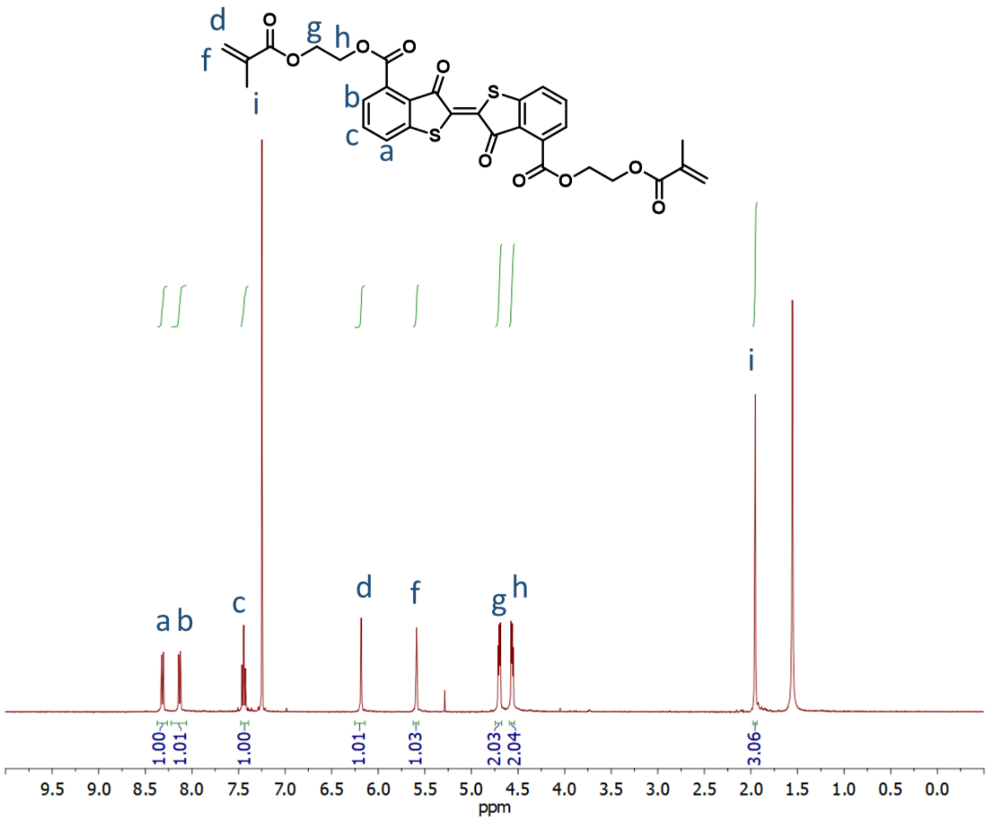

Supplementary Figure 9. ^{1}H NMR spectrum of *trans*-**1** ($CDCl_3$, 400 MHz).

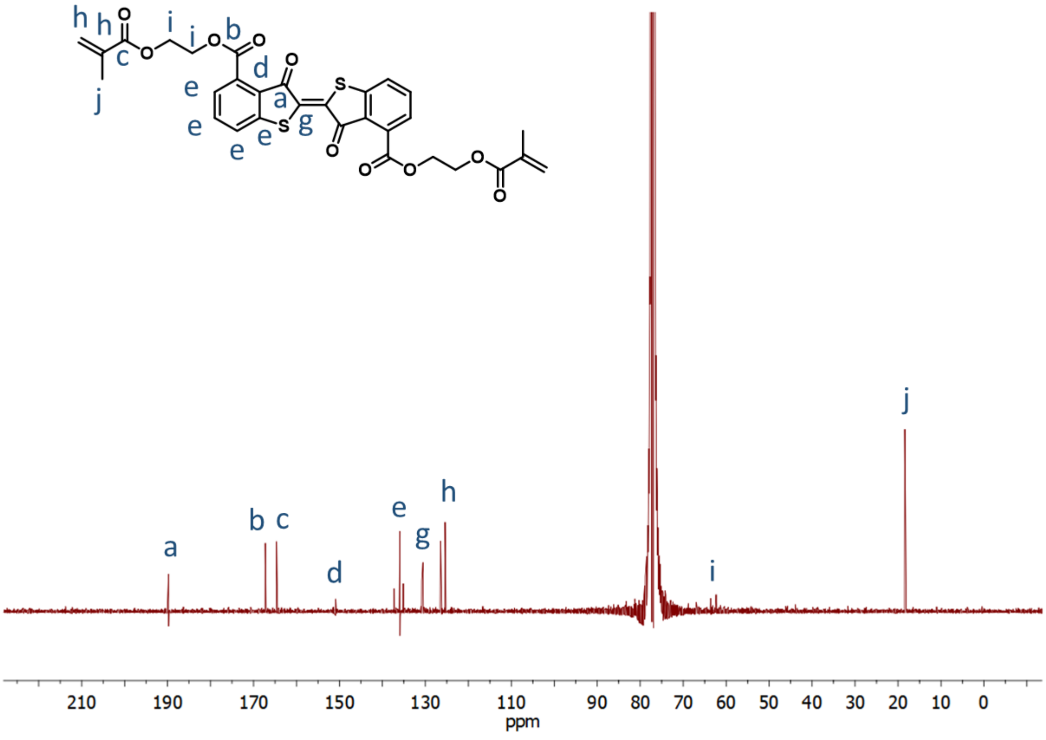

Supplementary Figure 10. ^{13}C NMR spectrum of *trans*-**1** ($CDCl_3$, 400 MHz).

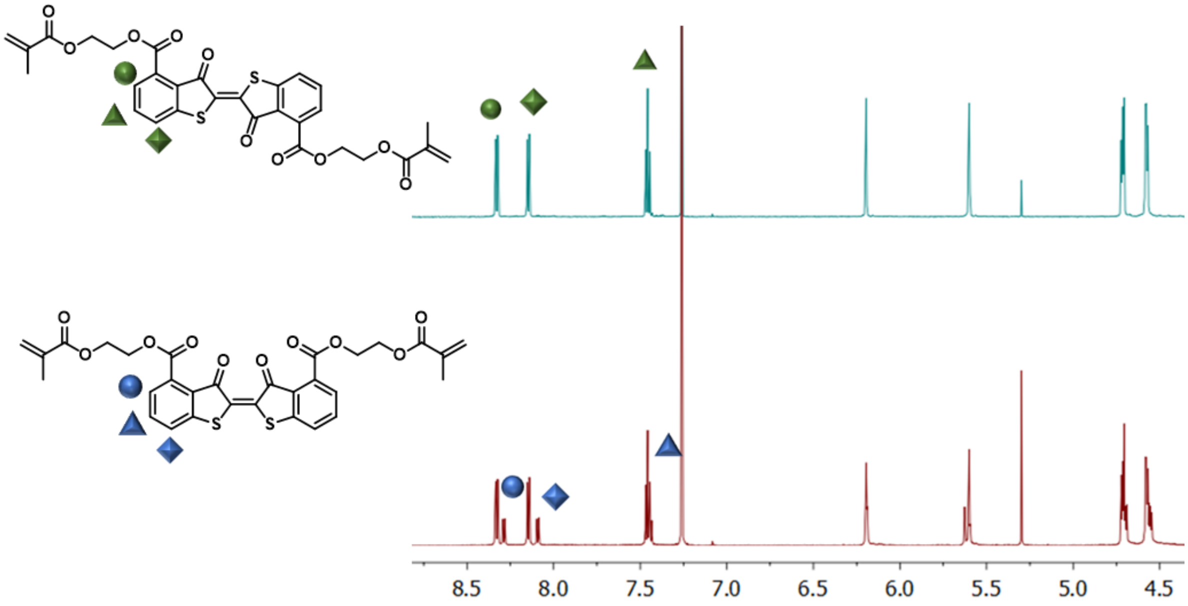

Supplementary Figure 11. ^{1}H NMR spectra of *trans*-**1** in $CDCl_3$ (400 MHz) before and after irradiation with an LED green light (λmax = 540 nm), showing the partial conversion to *cis*-**1**.

Synthesis of PEG_{44}-$(SH)_2$

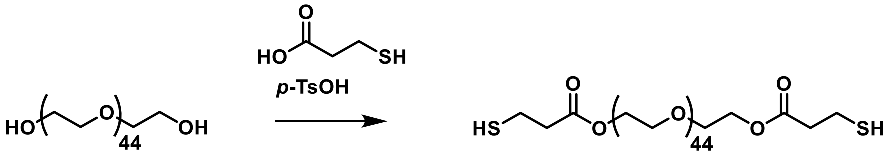

PEG_{44}-$(OH)_2$ (5 g, 2.5 mmol), mercaptopropionic acid (1.08 g, 10 mmol), and *para*-toluene sulfonic acid (10 mg, catalytic amount) were added to a solution of cyclohexane (100 mL) in a 250 mL round-bottom flask. The solution was heated at 100 °C under Dean–Stark conditions for 14 h and cooled to ambient temperature. Subsequently, it was concentrated in vacuo and the residue was dissolved in dichloromethane (50 mL), washed with saturated $NaHCO_3$ solution (50 mL), water (50 mL), and brine (50 mL). The organic layer was dried ($MgSO_4$), concentrated to ca. 5 mL, and precipitated by dropwise addition in diethyl ether (200 mL). The polymer product PEG_{44}-$(SH)_2$ was collected as white powder (yield: 4.9 g, 81.7%).

^{1}H NMR (500 MHz, $CDCl_3$, δ, ppm): 4.24–4.26 (t, J = 4.8 Hz), 3.61 (broad s), 2.75–2.77 (q, J = 8.4, 7.3 Hz), 2.69 (t, J = 7 Hz). NMR data are in agreement with the previously reported PEG_{44}-$(SH)_2$.[1]

Synthesis of 4arm PEG_{448}-(propiolate)$_4$

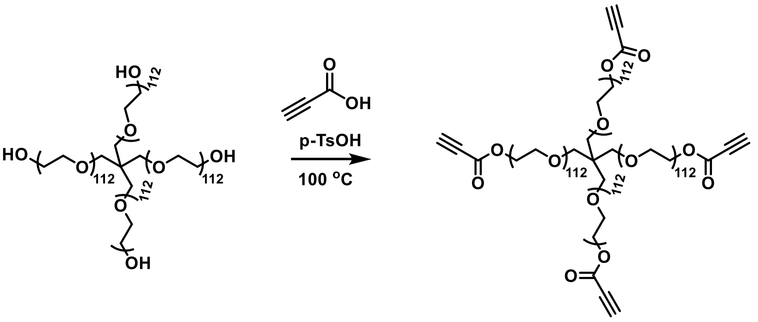

PEG_{448}-$(OH)_4$ (4 g, 0.2 mmol), propiolic acid (0.28 g, 4 mmol), and *para*-toluene sulfonic acid (5 mg, catalytic amount) were added to a solution of cyclohexane (100 mL) in a 250 mL round-bottom flask. The solution was heated at 100 °C under Dean–Stark conditions for 20 h and cooled to ambient temperature. Subsequently, it was concentrated in vacuo and the residue was dissolved in dichloromethane (50 mL), washed with water (50 mL), and brine (50 mL). The organic layer was dried ($MgSO_4$), concentrated to ca. 10 mL, and precipitated by dropwise addition into diethyl ether (200 mL). The polymer product PEG_{448}-(propiolate)$_4$ was collected as white powder (yield: 3.5 g, 87.5%).

^{1}H NMR (δ, ppm): 4.34-4.36 (t, *J* = 4.8 Hz), 3.66 (broad s), 3.65 (s), 3.0 (s). NMR data are in agreement with the previously reported 4arm PEG_{448}-(propiolate)$_4$.[2]

Synthesis of PEG-thioindigo (P1)

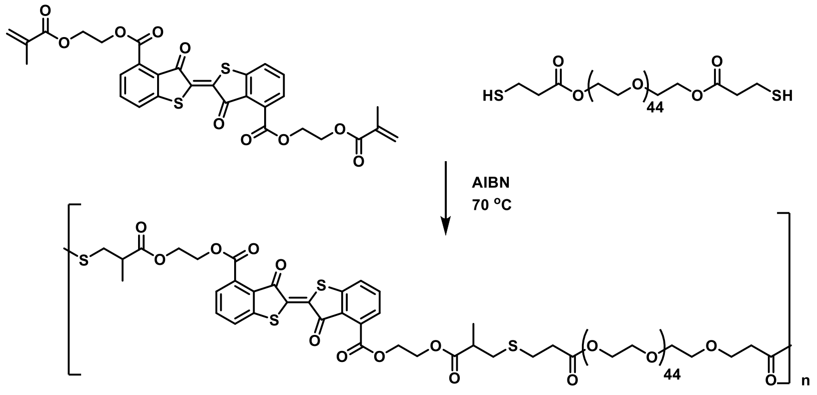

PEG_{44}-$(SH)_2$ (0.5 g, 0.24 mmol) and *trans*-1 (144 mg, 0.24 mmol) were dissolved in $CHCl_3$ (200 mL) in a 500 mL round bottom flask. AIBN (5 mg, catalytic amount) was added to the solution and the solution was heated at 70 °C under reflux for 24 h. The solution was concentrated *in vacuo* to ca. 10 mL and added dropwise into a solution of diethyl ether (200 mL). The product was collected as deep red powder (yield: 0.59 g, 91.61%).

^{1}H NMR (δ, ppm): 8.67 (broad s), 8.21 (broad s), 7.42 (broad s), 4.5-4.61 (broad m) 4.24 (s), 3.47 (m), 3 (m), 2.85 (m), 2.71 (m), 1.57 (m), 1.2-1.4 (broad m).

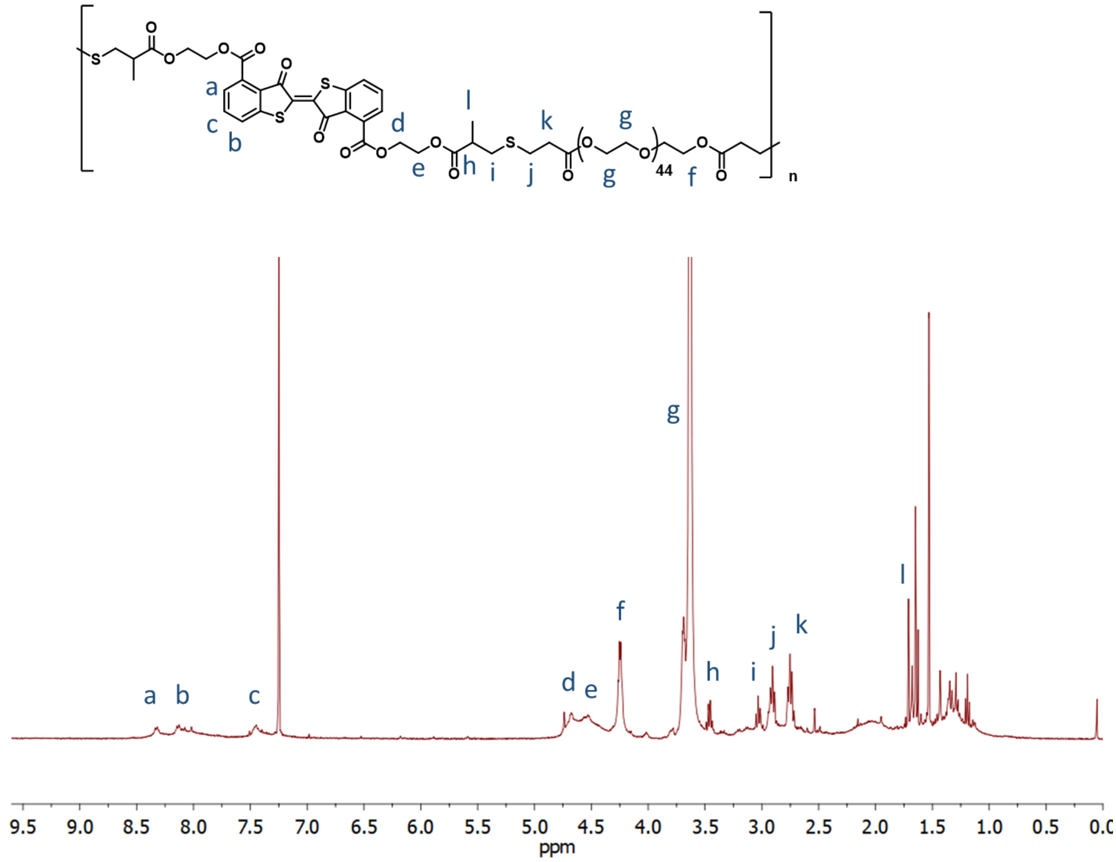

Supplementary Figure 12. ^{1}H NMR spectrum of **P1** ($CDCl_3$, 400 MHz).

2. Supplementary Figures

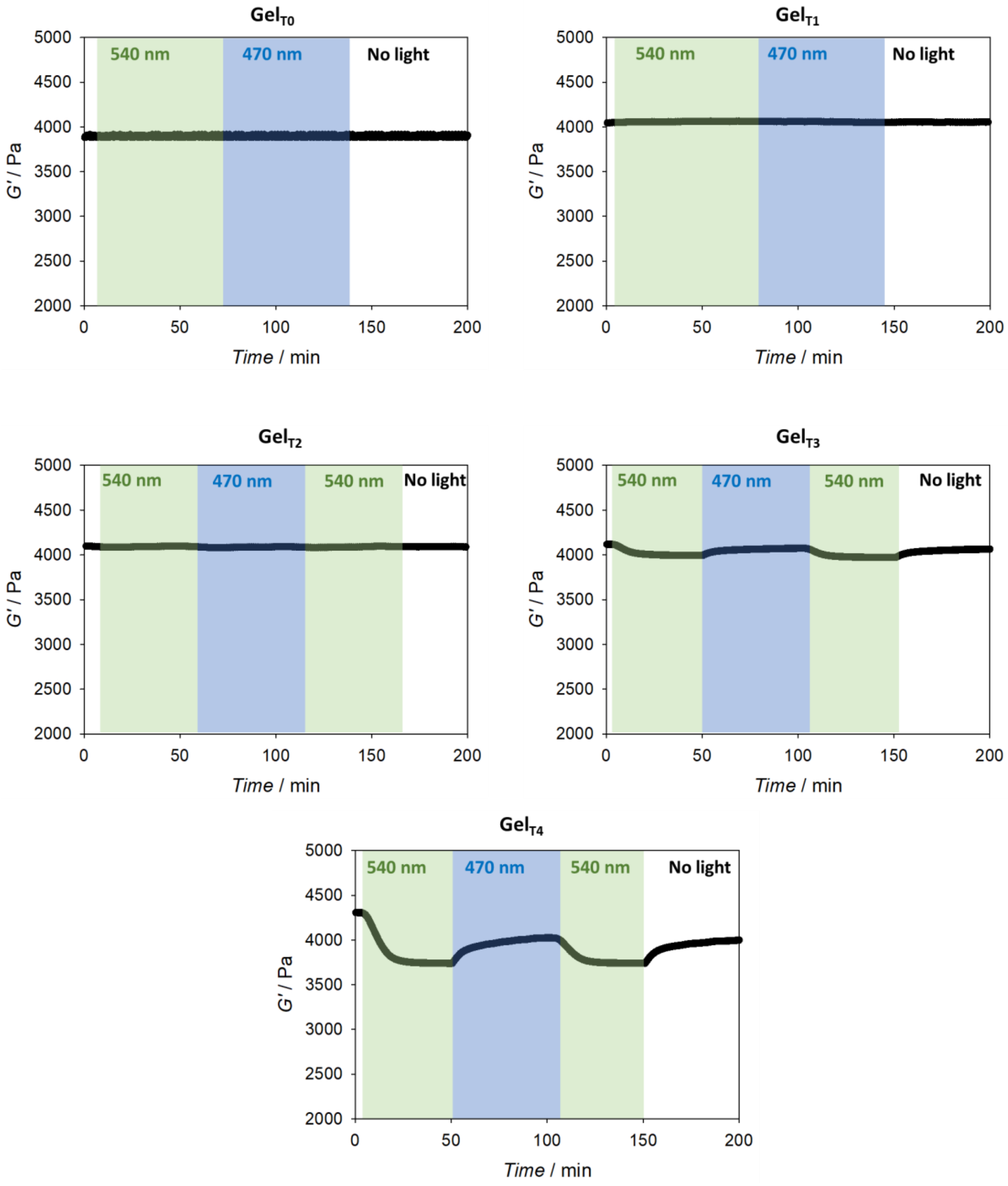

Supplementary Figure 13. Rheological data of the photoswitching of **Gel$_{T0}$-Gel$_{T4}$** under alternating green and blue light and green light irradiation.

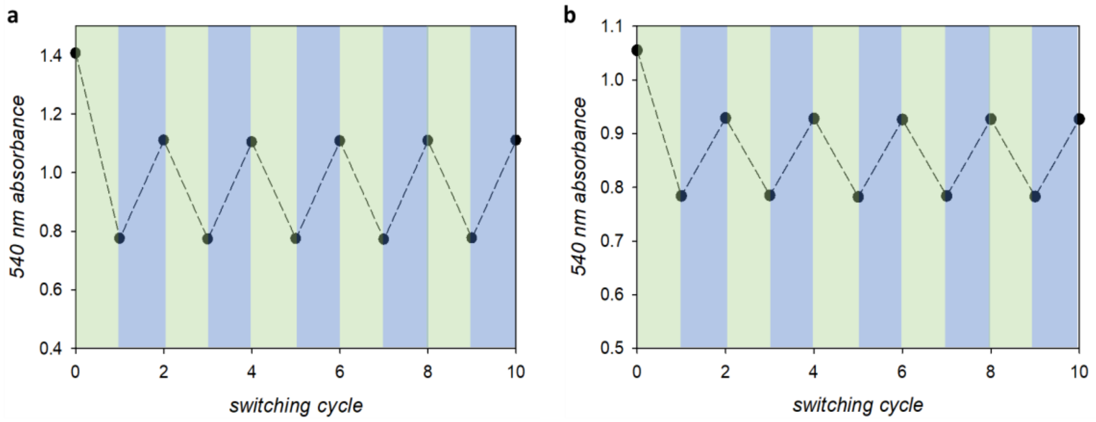

Supplementary Figure 14. Cyclic photoswitching of UV-Vis absorbance **P1** solution in **a)** methanol and **b)** water by the change in 540 nm absorbance.

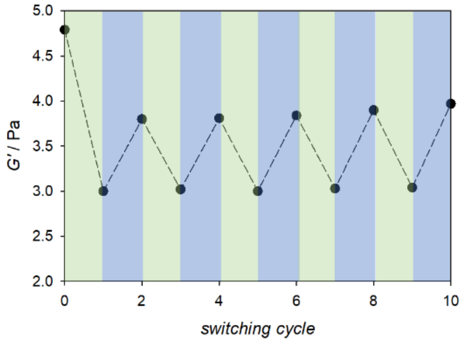

Supplementary Figure 15. Cyclic photoswitching of storage modulus G' of **GelT$_5$** hydrogel.

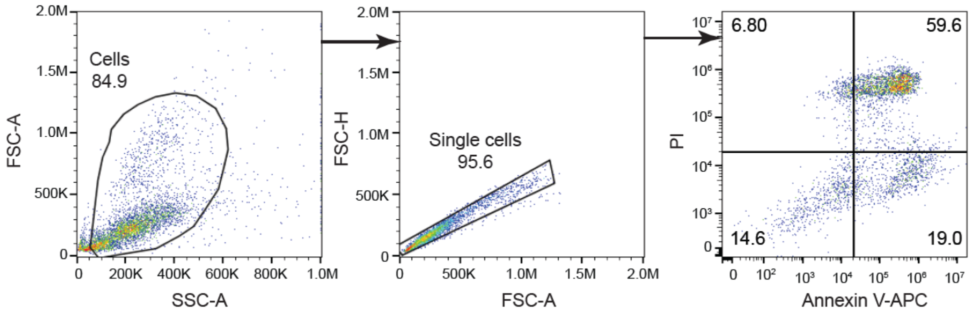

Supplementary Figure 16. Workflow for the gating strategy for flow cytometric data. FSC-A versus SSC-A plot was used to gate for cells. Next, single cells were gated based on FSC-width versus FSC-height, and then live cells, apoptotic and necrotic cells were gated using PI and Annexin-APC: viable cells (Annexin-PI-), early apoptotic cells (Annexin+PI-), late apoptotic cells (Annexin+PI+) and necrotic cells (Annexin-PI+).

3. Supplementary Tables

Adjusted p-values for the comparison of the proportions of viable, early apoptotic, late apoptotic and necrotic cells after 24 h treatment with hydrogels, or with DMSO as determined by one-way ANOVA test with Tukey post-hoc test. Significant differences are shown in bold.

HEK293T cells	Live (Annexin$^-$PI$^-$)	Early apoptotic (Annexin$^+$PI$^-$)	Late apoptotic (Annexin$^+$PI$^+$)	Necrotic (Annexin$^-$PI$^+$)
Untreated vs. Non-irridiated gel	0.9620	0.9850	>0.9999	0.9637
Untreated vs. Irridiated gel	0.9960	0.9938	>0.9999	0.9995
Untreated vs. DMSO	**<0.0001**	**<0.0001**	**<0.0001**	0.9995
Non-irridiated gel vs. Irridiated gel	0.9935	0.9997	>0.9999	0.9826
Non-irridiated gel vs. DMSO	**<0.0001**	**<0.0001**	**<0.0001**	0.9355
Irridiated gel vs. DMSO	**<0.0001**	**<0.0001**	**<0.0001**	0.9961

PBMCs	Live (Annexin$^-$PI$^-$)	Early apoptotic (Annexin$^+$PI$^-$)	Late apoptotic (Annexin$^+$PI$^+$)	Necrotic (Annexin$^-$PI$^+$)
Untreated vs. Non-irridiated gel	0.9996	>0.9999	0.9995	0.9941
Untreated vs. Irridiated gel	0.9387	0.9989	0.9994	0.7242
Untreated vs. DMSO	**<0.0001**	0.8901	**<0.0001**	**<0.0001**
Non-irridiated gel vs. Irridiated gel	0.9633	0.9997	>0.9999	0.8504
Non-irridiated gel vs. DMSO	**<0.0001**	0.9076	**<0.0001**	**<0.0001**
Irridiated gel vs. DMSO	**<0.0001**	0.9386	**<0.0001**	**<0.0001**

4. Supplementary References

1. Truong, V. X.; Tsang, K. M.; Forsythe, J. S., Nonswelling Click-Cross-Linked Gelatin and PEG Hydrogels with Tunable Properties Using Pluronic Linkers. *Biomacromolecules* **2017,** *18* (3), 757-766.
2. Li, M.; Dove, A. P.; Truong, V. X., Additive-Free Green Light-Induced Ligation Using BODIPY Triggers. *Angewandte Chemie International Edition* **2020,** *59* (6), 2284-2288.
