# Supplementary material for: Visible light-induced switching of soft matter materials properties based on thioindigo photoswitches
Source: Nat Commun. 2023 Dec 14;14:8298. doi: 10.1038/s41467-023-44128-8 (PMC10721821; doi:10.1038/s41467-023-44128-8)
Supplement: Supplementary file 3 — Description of Additional Supplementary Files [file 41467_2023_44128_MOESM3_ESM.pdf]

Description of Additional Supplementary Files

File Name: Supplementary Data 1
Description: Atomic coordinates of selected molecules calculated based on DFT/TD-DFT methods.
