# Supplementary material for: Visible light-induced switching of soft matter materials properties based on thioindigo photoswitches
Source: Nat Commun. 2023 Dec 14;14:8298. doi: 10.1038/s41467-023-44128-8 (PMC10721821; doi:10.1038/s41467-023-44128-8)
Supplement: Supplementary file 5 — Reporting Summary [file 41467_2023_44128_MOESM5_ESM.pdf]

nature portfolio

Corresponding author(s): Xian Jun Loh, Christopher Barner-Kowollik, Vinh X. Truong

Last updated by author(s): Nov 20, 2023

Reporting Summary

Nature Portfolio wishes to improve the reproducibility of the work that we publish. This form provides structure for consistency and transparency in reporting. For further information on Nature Portfolio policies, see our Editorial Policies and the Editorial Policy Checklist.

Statistics

For all statistical analyses, confirm that the following items are present in the figure legend, table legend, main text, or Methods section.

n/a	Confirmed	
☐	☒	The exact sample size (*n*) for each experimental group/condition, given as a discrete number and unit of measurement
☐	☒	A statement on whether measurements were taken from distinct samples or whether the same sample was measured repeatedly
☐	☒	The statistical test(s) used AND whether they are one- or two-sided<br>*Only common tests should be described solely by name; describe more complex techniques in the Methods section.*
☒	☐	A description of all covariates tested
☐	☒	A description of any assumptions or corrections, such as tests of normality and adjustment for multiple comparisons
☐	☒	A full description of the statistical parameters including central tendency (e.g. means) or other basic estimates (e.g. regression coefficient) AND variation (e.g. standard deviation) or associated estimates of uncertainty (e.g. confidence intervals)
☐	☒	For null hypothesis testing, the test statistic (e.g. *F*, *t*, *r*) with confidence intervals, effect sizes, degrees of freedom and *P* value noted<br>*Give P values as exact values whenever suitable.*
☒	☐	For Bayesian analysis, information on the choice of priors and Markov chain Monte Carlo settings
☒	☐	For hierarchical and complex designs, identification of the appropriate level for tests and full reporting of outcomes
☒	☐	Estimates of effect sizes (e.g. Cohen's *d*, Pearson's *r*), indicating how they were calculated

Our web collection on statistics for biologists contains articles on many of the points above.

Software and code

Policy information about availability of computer code

Data collection	No software was used for data collection.
Data analysis	MestReNova, SigmaPlot v15, OriginPro, FlowJo v10, GraphPad Prism 8

For manuscripts utilizing custom algorithms or software that are central to the research but not yet described in published literature, software must be made available to editors and reviewers. We strongly encourage code deposition in a community repository (e.g. GitHub). See the Nature Portfolio guidelines for submitting code & software for further information.

Data

Policy information about availability of data

All manuscripts must include a data availability statement. This statement should provide the following information, where applicable:

- Accession codes, unique identifiers, or web links for publicly available datasets
- A description of any restrictions on data availability
- For clinical datasets or third party data, please ensure that the statement adheres to our policy

The authors declare that the data supporting the findings of this study are available within the article and its Supplementary Information file.

Research involving human participants, their data, or biological material

Policy information about studies with human participants or human data. See also policy information about sex, gender (identity/presentation), and sexual orientation and race, ethnicity and racism.

Reporting on sex and gender	N.A.
Reporting on race, ethnicity, or other socially relevant groupings	N.A.
Population characteristics	N.A.
Recruitment	N.A.
Ethics oversight	N.A.

Note that full information on the approval of the study protocol must also be provided in the manuscript.

Field-specific reporting

Please select the one below that is the best fit for your research. If you are not sure, read the appropriate sections before making your selection.

[x] Life sciences [ ] Behavioural & social sciences [ ] Ecological, evolutionary & environmental sciences

For a reference copy of the document with all sections, see nature.com/documents/nr-reporting-summary-flat.pdf

Life sciences study design

All studies must disclose on these points even when the disclosure is negative.

Sample size	Sample size used for in vitro cell cytotoxicity testing was determined using the software G*Power 3.1 for one-way ANOVA test to compare mean difference of four independent groups with effect size f=1.6; α err prob=0.05 and power=0.95.
Data exclusions	No data were excluded from the analyses.
Replication	Each experiment was performed in general biological triplicates. All attempts at replication were successful.
Randomization	Cells were spit randomly for different treatments.
Blinding	No blinding experiments are included, as no human or animals are involved in the studies. The scientist undertaking the cell experiments were not aware of, or expected any possible outcomes from the cell culture experiments.

Reporting for specific materials, systems and methods

We require information from authors about some types of materials, experimental systems and methods used in many studies. Here, indicate whether each material, system or method listed is relevant to your study. If you are not sure if a list item applies to your research, read the appropriate section before selecting a response.

Materials & experimental systems

n/a	Involved in the study	
x		Antibodies
	x	Eukaryotic cell lines
x		Palaeontology and archaeology
x		Animals and other organisms
x		Clinical data
x		Dual use research of concern
x		Plants

Methods

n/a	Involved in the study	
x		ChIP-seq
	x	Flow cytometry
x		MRI-based neuroimaging

Eukaryotic cell lines

Policy information about cell lines and Sex and Gender in Research

Cell line source(s)	The human embryonic kidney (HEK-293T) cell line obtained from the American Type Culture Collection (ATCC, USA).
Authentication	The HEK-293T cell line was authenticated by the vendor.
Mycoplasma contamination	The HEK-293T was tested negative for mycoplasma contamination.
Commonly misidentified lines (See ICLAC register)	No commonly misidentified cell line was used.

Plants

Seed stocks	N.A.
Novel plant genotypes	N.A.
Authentication	N.A.

Flow Cytometry

Plots

Confirm that:

- [x] The axis labels state the marker and fluorochrome used (e.g. CD4-FITC).
- [x] The axis scales are clearly visible. Include numbers along axes only for bottom left plot of group (a 'group' is an analysis of identical markers).
- [x] All plots are contour plots with outliers or pseudocolor plots.
- [x] A numerical value for number of cells or percentage (with statistics) is provided.

Methodology

Sample preparation	After treatments, cells were harvested and washed in cold Annexin V binding buffer (BD Pharmingen, USA), followed by incubation with 100 µL Annexin V binding buffer containing 5 µL of APC Annexin V-APC (Biolegend, USA) and 1 µL of 100 µg/mL Propodium Iodide (PI, (ThermoFisher Scientific) working solution for 15 min at room temperature in the dark. Then cells were washed with Annexin V binding buffer, and samples were acquired by flow cytometry.
Instrument	CytoFLEX LX (Beckman Coulter, USA)
Software	The flow cytometric data were analyzed using the FlowJo software V10 (Three Star, USA) and GraphPad Prism 8 (San Diego,CA, USA).
Cell population abundance	Approximately 50,000 - 100,000 cells were stained and acquired for flow cytometry.
Gating strategy	FSC-A versus SSC-A plot was used to gate for cells. Next, single cells were gated based on FSC-width versus FSC-height, and then live cells, apoptotic and necrotic cells were gated using PI and Annexin-APC as indicated in the Figure legend.

- [x] Tick this box to confirm that a figure exemplifying the gating strategy is provided in the Supplementary Information.
